# Gene–diet interaction between *CETP* rs3764261 and vegetarian diet on HDL-C levels: evidence for precision nutrition from the Taiwan Biobank

**DOI:** 10.3389/fnut.2026.1893336

**Published:** 2026-08-05

**Authors:** Ying-Hsiang Chou, Yeu-Sheng Tyan, Shih-Tsung Chang, Oswald Ndi Nfor, Wen-Yu Lu, Yi-Ching Liaw, Yung-Po Liaw

**Affiliations:** 1 Department of Radiation Oncology, Chung Shan Medical University Hospital, Taichung, Taiwan; 2 School of Medicine, Chung Shan Medical University, Taichung, Taiwan; 3 School of Medical Imaging and Radiological Sciences, Chung Shan Medical University, Taichung, Taiwan; 4 Department of Medical Imaging, Chung Shan Medical University Hospital, Taichung, Taiwan; 5 Institute of Medicine, Chung Shan Medical University, Taichung, Taiwan; 6 Department of Public Health and Institute of Public Health, Chung Shan Medical University, Taichung, Taiwan; 7 Department of Nutrition, China Medical University, Taichung, Taiwan

**Keywords:** *CETP* rs3764261, gene–diet interaction, HDL-C, precision nutrition, vegetarian diet

## Abstract

**Background:**

Precision nutrition seeks genotype-informed dietary guidance. We examined the associations of *CETP* rs3764261 and a vegetarian diet with high-density lipoprotein cholesterol (HDL-C) levels and the potential modification of this association by the variant in Taiwanese adults.

**Methods:**

We analyzed 9,263 adults from the Taiwan Biobank with rs3764261 genotype data and available HDL-C measurements. Rs3764261 genotypes were obtained from whole-genome genotyping using the TWBv2.0 Affymetrix SNP array, with quality control based on Hardy–Weinberg equilibrium, minor allele frequency, and call-rate thresholds. Vegetarian diet status was defined using questionnaire data (being vegetarian throughout the day for >6 months). Associations were evaluated using multiple linear regression models, adjusting for relevant covariates and testing the rs3764261 × vegetarian-diet interaction.

**Results:**

A total of 9,263 adults were included, comprising 8,330 non-vegetarians and 933 vegetarians. Vegetarians had significantly lower mean HDL-C than non-vegetarians (53.190 vs. 56.031 mg/dL, *p* < 0.0001) and lower LDL-C (*p* < 0.0001). The distribution of *CETP* rs3764261 genotypes did not differ by diet (*p* = 0.8413). In multivariable analysis, rs3764261 CA (*β* = 3.3318, *p* < 0.0001) and AA (*β* = 8.4651, *p* < 0.0001) genotypes were associated with higher HDL-C compared with CC. However, a vegetarian diet was associated with lower HDL-C (*β* = −3.4040, *p* < 0.0001). A significant interaction between rs3764261 and vegetarian diet was observed (*p* for interaction = 0.0045). The positive association of CA and AA genotypes with HDL-C was evident among non-vegetarians, but not vegetarians, and the HDL-lowering effect of a vegetarian diet became progressively stronger across the CC, CA, and AA genotypes (*β* = −2.6779, −4.8479, and −8.1884 mg/dL, respectively). However, the small number of AA homozygotes among vegetarians (*n* = 21) limited statistical power for some stratified estimates, and these findings warrant replication in independent cohorts.

**Conclusion:**

Rs3764261 modified the association between vegetarian diet status and HDL-C levels, supporting evidence for gene–diet interactions in precision nutrition approaches to lipid risk management.

## 1 Introduction

High-density lipoprotein cholesterol (HDL-C) has long been inversely associated with atherosclerotic cardiovascular disease (ASCVD) risk and is involved in reverse cholesterol transport (1, 2). Both genetic and environmental factors contribute substantially to inter-individual variation in HDL-C levels, with heritability estimates ranging from 40% to 60% (3).

The cholesteryl ester transfer protein (*CETP*) gene is a major genetic determinant of HDL-C levels identified through genome-wide association studies (4, 5). *CETP* mediates the transfer of cholesteryl esters from HDL particles to apolipoprotein B-containing lipoproteins, thereby influencing HDL metabolism and lipid transport (6). The rs3764261 polymorphism is located approximately 2.5 kb upstream of *CETP*, in the 5′ flanking region, and has been repeatedly associated with HDL-C levels in population-based studies; in Taiwanese data, the A allele has been associated with higher HDL-C concentrations (7). Although functional studies support the biological relevance of *CETP* expression and activity in determining HDL-C levels, direct functional evidence for rs3764261-specific effects remains limited (6, 8).

Although vegetarian dietary patterns are generally associated with favorable reductions in total cholesterol and low-density lipoprotein cholesterol (LDL-C) levels, their effects on HDL-C have remained inconsistent across studies and populations (9–12). Some individuals exhibit substantial reductions in HDL-C, whereas others show minimal changes, suggesting that inter-individual biological heterogeneity may influence lipid responses to plant-based diets. The mechanisms underlying this variability remain poorly understood, but genetic factors involved in HDL metabolism may partially explain these differential responses. Potential mechanisms may involve differences in dietary fatty acid composition, cholesterol intake, fiber and plant sterol intake, and lipid metabolism pathways, including gut microbiota-related metabolic changes (13, 14).

Gene–diet interactions are increasingly relevant to precision nutrition research because genetic background may influence metabolic responses to dietary patterns (15–17). Recent systematic reviews have discussed the potential value of incorporating lipid-related genetic variants into personalized dietary recommendations, while also emphasizing the need for stronger population-specific evidence (16, 17). A previous Taiwan Biobank study evaluated the combined associations of exercise and vegetarian diet with HDL-C according to *MTHFR* rs1801133, but whether *CETP* rs3764261 modifies the relationship between vegetarian diet and HDL-C remains unclear (18).

Taiwan provides a useful setting for investigating gene–diet interactions because vegetarian dietary practices are relatively common and the Taiwan Biobank provides integrated genomic, biochemical, and lifestyle data for population-based biomedical research (19). Given the increasing clinical interest in personalized dietary recommendations, identifying genetic modifiers of HDL-C responses to vegetarian diets may have implications for precision nutrition and individualized cardiometabolic risk management in East Asian populations.

Therefore, this study aimed to investigate the associations of *CETP* rs3764261 and a vegetarian diet with HDL-C levels and to examine whether *CETP* rs3764261 modifies the relationship between a vegetarian diet and HDL-C concentrations in Taiwanese adults.

## 2 Materials and methods

### 2.1 Study population

The data were obtained from the Taiwan Biobank, which is a large-scale, population-based research database aimed at collecting health-related data and biological samples from the Taiwanese population. The Taiwan Biobank includes participants aged 30–70 years who are Taiwanese residents without a history of cancer. After signing informed consent, data collection included personal information, lifestyle details, and biological samples. A total of 9,263 participants with genotyping data were included in the analysis after excluding those with missing HDL-C values or incomplete questionnaire data.

### 2.2 Polymorphism information

The genotypes for rs3764261 were obtained through whole-genome genotyping from the Taiwan Biobank. The single nucleotide polymorphism (SNP) chip was TWBv2.0 from Affymetrix using the Axiom™ Genome-Wide Array Plate System (Affymetrix, Santa Clara, CA, USA). The rs3764261 SNP met the following quality control criteria: (1) *p*-value for Hardy–Weinberg equilibrium ≥ 0.000001; (2) minor allele frequency (MAF) ≥ 0.001; and (3) call rate ≥ 95%.

### 2.3 Variable definitions

Vegetarian diet status was defined based on the Taiwan Biobank questionnaire: individuals who consumed a vegetarian diet throughout the day and for more than half a year were classified as vegetarians. HDL-C levels were measured from blood samples collected during participant enrollment. The covariates included gender, age, cigarette smoking, alcohol intake, exercise habit, BMI, waist-hip ratio, hypertension, and low-density lipoprotein cholesterol (LDL-C).

### 2.4 Statistical analysis

Data were managed and analyzed using SAS 9.4 (SAS Institute Inc., Cary, NC, USA) and PLINK version 1.9. Demographic characteristics were analyzed using the chi-square test and Student’s *t*-test. The chi-square test was used to assess differences between vegetarian diet status and categorical variables; results are presented as numbers and percentages. Student’s *t*-test was used to assess differences between vegetarian diet status and continuous variables; results are presented as mean ± standard error. Multiple linear regression models were used to examine the associations between rs3764261, vegetarian diets, and HDL-C levels. Because this study evaluated a single *a priori*-selected candidate variant (rs3764261) and one pre-specified gene–diet interaction hypothesis, genome-wide multiple-testing correction was not applicable; for the two primary genotype contrasts (CA vs. CC and AA vs. CC) and the interaction term, the principal findings remained statistically significant after a conservative Bonferroni adjustment.

## 3 Results

Table 1 summarizes the characteristics of the 8,330 non-vegetarians and 933 vegetarians. Compared with non-vegetarians, vegetarians had significantly lower HDL-C and LDL-C (both *p* < 0.0001) and a higher proportion of women (*p* = 0.0003). Vegetarians were also less likely to smoke (*p* = 0.0039), to consume alcohol (*p* = 0.0001), and to exercise regularly (*p* = 0.0099). The distribution of rs3764261 genotypes did not differ by diet (*p* = 0.8413), and age, BMI, waist–hip ratio, and hypertension were comparable between groups (all *p* > 0.05).

**Table 1:** Demographic characteristics of the study participants stratified by vegetarian diet status.

Variables	No vegetarian diet (*n* = 8,330)	Vegetarian diet (*n* = 933)	*p*-value
HDL-C, mg/dL (mean ± SE)	56.031 ± 0.151	53.190 ± 0.424	<0.0001
rs3764261, *n* (%)			0.8413
CC	5,832 (70.01)	652 (69.88)	
CA	2,286 (27.44)	260 (27.87)	
AA	212 (2.55)	21 (2.25)	
Gender, *n* (%)			0.0003
Women	6,246 (74.98)	750 (80.39)	
Men	2,084 (25.02)	183 (19.61)	
Age, years (mean ± SE)	50.351 ± 0.112	50.075 ± 0.316	0.4108
Cigarette smoking, *n* (%)			0.0039
No	7,081 (85.01)	826 (88.53)	
Yes	1,249 (14.99)	107 (11.47)	
Alcohol consumption, *n* (%)			0.0001
No	7,788 (93.49)	902 (96.68)	
Yes	542 (6.51)	31 (3.32)	
Exercise habit, *n* (%)			0.0099
No	4,686 (56.25)	566 (60.66)	
Yes	3,644 (43.75)	367 (39.34)	
BMI categories, *n* (%)			0.1385
Underweight (BMI < 18.5 kg/m^2^)	257 (3.09)	40 (4.29)	
Normal weight (18.5 ≤ BMI < 24 kg/m^2^)	4,339 (52.09)	497 (53.27)	
Overweight (24 ≤ BMI < 27 kg/m^2^)	2,236 (26.84)	244 (26.15)	
Obesity (BMI ≥ 27 kg/m^2^)	1,498 (17.98)	152 (16.29)	
Waist-hip ratio, *n* (%)			0.7218
Normal (men ≤ 0.9; women ≤ 0.85)	4,417 (53.03)	489 (52.41)	
Abnormal (men > 0.9; women > 0.85)	3,913 (46.97)	444 (47.59)	
Hypertension, *n* (%)			0.5387
No	7,372 (88.50)	832 (89.17)	
Yes	958 (11.50)	101 (10.83)	
LDL-C, mg/dL (mean ± SE)	121.200 ± 0.344	115.500 ± 0.997	<0.0001

BMI, body mass index; HDL-C, high-density lipoprotein cholesterol; LDL-C, low-density lipoprotein cholesterol; *n*, sample size; SE, standard error.

Table 2 presents the association between rs3764261, vegetarian diet, and HDL-C levels. The interaction between rs3764261 genotype and vegetarian diet was statistically significant (*p* = 0.0045). Compared with the CC genotype, the CA (*β* = 3.3318, *p* < 0.0001) and AA (*β* = 8.4651, *p* < 0.0001) genotypes were significantly associated with higher HDL-C levels. Vegetarian diet was associated with lower HDL-C levels compared with a non-vegetarian diet (*β* = −3.4040, *p* < 0.0001).

**Table 2:** Association of rs3764261, vegetarian diet, and HDL-C levels.

Variables	*β*	95% CI lower	95% CI upper	*p*-value
rs3764261				
CC (ref)	—	—	—	—
CA	3.3318	2.7909	3.8727	<0.0001
AA	8.4651	6.9231	10.0071	<0.0001
Vegetarian diet				
No (ref)	—	—	—	—
Yes	−3.4040	−4.2044	−2.6035	<0.0001
Gender				
Women (ref)	—	—	—	—
Men	−8.6158	−9.2810	−7.9506	<0.0001
Age, years	0.1091	0.0822	0.1361	<0.0001
Cigarette smoking				
No (ref)	—	—	—	—
Yes	−1.8506	−2.6577	−1.0436	<0.0001
Alcohol consumption				
No (ref)	—	—	—	—
Yes	2.8067	1.7350	3.8783	<0.0001
Exercise habit				
No (ref)	—	—	—	—
Yes	1.2710	0.7541	1.7880	<0.0001
BMI categories				
Normal weight (ref)	—	—	—	—
Underweight	7.0913	5.6994	8.4832	<0.0001
Overweight	−4.8627	−5.4567	−4.2686	<0.0001
Obesity	−7.1020	−7.8180	−6.3859	<0.0001
Waist-hip ratio				
Normal (ref)	—	—	—	—
Abnormal	−4.3061	−4.8373	−3.7750	<0.0001
Hypertension				
No (ref)	—	—	—	—
Yes	−2.3059	−3.1069	−1.5050	<0.0001
LDL-C	0.0018	−0.0061	0.0097	0.6543

Interaction of rs3764261 × vegetarian diet: *p* = 0.0045.

*β*, regression coefficient; BMI, body mass index; CI, confidence interval; HDL-C, high-density lipoprotein cholesterol; LDL-C, low-density lipoprotein cholesterol; ref, reference group.

Table 3 shows the association between the rs3764261 genotype and HDL-C levels, stratified by vegetarian diet status. Among non-vegetarians, the CA (*β* = 3.5414, *p* < 0.0001) and AA (*β* = 8.9378, *p* < 0.0001) genotypes were significantly associated with higher HDL-C levels, with CC as the reference. However, no significant association was found in the vegetarian group.

**Table 3:** Association between rs3764261 and HDL-C levels, stratified by vegetarian diet status.

Variables	Non-vegetarians (*n* = 8,330)				Vegetarians (*n* = 933)			
	*β*	95% CI lower	95% CI upper	*p*-value	*β*	95% CI lower	95% CI upper	*p*-value
rs3764261								
CC (ref)	—	—	—	—	—	—	—	—
CA	3.5414	2.9690	4.1138	<0.0001	1.3633	−0.2977	3.0244	0.1076
AA	8.9378	7.3156	10.5600	<0.0001	3.4692	−1.5267	8.4651	0.1733
Gender								
Women (ref)	—	—	—	—	—	—	—	—
Men	−8.7454	−9.4457	−8.0451	<0.0001	−7.2080	−9.3552	−5.0608	<0.0001
Age, years	0.1153	0.0869	0.1437	<0.0001	0.0641	−0.0219	0.1501	0.1439
Cigarette smoking								
No (ref)	—	—	—	—	—	—	—	—
Yes	−1.7960	−2.6444	−0.9475	<0.0001	−2.2416	−4.8818	0.3986	0.0960
Alcohol consumption								
No (ref)	—	—	—	—	—	—	—	—
Yes	2.6854	1.5768	3.7940	<0.0001	4.7789	0.4277	9.1302	0.0314
Exercise habit								
No (ref)	—	—	—	—	—	—	—	—
Yes	1.1698	0.6222	1.7174	<0.0001	1.9680	0.3996	3.5363	0.0140
BMI categories								
Normal weight (ref)	—	—	—	—	—	—	—	—
Underweight	6.8014	5.3027	8.3001	<0.0001	8.8877	5.1622	12.6132	<0.0001
Overweight	−4.8471	−5.4748	−4.2194	<0.0001	−5.2428	−7.0877	−3.3979	<0.0001
Obesity	−7.0924	−7.8458	−6.3389	<0.0001	−7.3618	−9.6770	−5.0466	<0.0001
Waist-hip ratio								
Normal (ref)	—	—	—	—	—	—	—	—
Abnormal	−4.4040	−4.9646	−3.8434	<0.0001	−3.3144	−4.9851	−1.6436	0.0001
Hypertension								
No (ref)	—	—	—	—	—	—	—	—
Yes	−2.2668	−3.1110	−1.4225	<0.0001	−2.8392	−5.3884	−0.2899	0.0291
LDL-C	−0.0006	−0.0089	0.0077	0.8916	0.0235	−0.0016	0.0485	0.0661

*β*, regression coefficient; BMI, body mass index; CI, confidence interval; HDL-C, high-density lipoprotein cholesterol; LDL-C, low-density lipoprotein cholesterol; ref, reference group.

Table 4 presents the association between vegetarian diet and HDL-C levels, stratified by rs3764261 genotype. Regardless of genotype, individuals consuming a vegetarian diet had significantly lower HDL-C levels compared with those consuming a non-vegetarian diet. The *β* for HDL-C was −2.6779 (*p* < 0.0001) in CC, −4.8479 (*p* < 0.0001) in CA, and −8.1884 (*p* = 0.0148) in AA genotypes.

**Table 4:** Association between vegetarian diet and HDL-C levels, stratified by rs3764261 genotype.

Variables	rs3764261 CC				rs3764261 CA				rs3764261 AA			
	*β*	95% CI lower	95% CI upper	*p*-value	*β*	95% CI lower	95% CI upper	*p*-value	*β*	95% CI lower	95% CI upper	*p*-value
Vegetarian diet												
No (ref)	—	—	—	—	—	—	—	—	—	—	—	—
Yes	−2.6779	−3.6025	−1.7533	<0.0001	−4.8479	−6.4637	−3.2321	<0.0001	−8.1884	−14.7595	−1.6173	0.0148
Gender												
Women (ref)	—	—	—	—	—	—	—	—	—	—	—	—
Men	−8.5763	−9.3447	−7.8079	<0.0001	−8.4634	−9.7999	−7.1270	<0.0001	−9.9099	−15.7467	−4.0732	0.0010
Age, years	0.1063	0.0754	0.1372	<0.0001	0.1035	0.0483	0.1588	0.0002	0.2698	0.0522	0.4874	0.0153
Cigarette smoking												
No (ref)	—	—	—	—	—	—	—	—	—	—	—	—
Yes	−2.0734	−2.9972	−1.1496	<0.0001	−1.1762	−2.8459	0.4936	0.1673	−3.1653	−9.6655	3.3349	0.3383
Alcohol consumption												
No (ref)	—	—	—	—	—	—	—	—	—	—	—	—
Yes	2.9090	1.6527	4.1653	<0.0001	2.7798	0.6959	4.8637	0.0090	−1.4198	−10.6983	7.8587	0.7633
Exercise habit												
No (ref)	—	—	—	—	—	—	—	—	—	—	—	—
Yes	1.4469	0.8519	2.0418	<0.0001	1.0023	−0.0540	2.0585	0.0629	−1.9253	−5.9627	2.1121	0.3483
BMI categories												
Normal weight (ref)	—	—	—	—	—	—	—	—	—	—	—	—
Underweight	7.4242	5.8637	8.9846	<0.0001	5.3491	2.3113	8.3868	0.0006	16.4564	4.4888	28.4239	0.0073
Overweight	−4.5824	−5.2654	−3.8994	<0.0001	−5.8751	−7.0901	−4.6602	<0.0001	−1.8807	−6.5735	2.8122	0.4305
Obesity	−7.0432	−7.8669	−6.2195	<0.0001	−7.2305	−8.6926	−5.7684	<0.0001	−8.0797	−13.8197	−2.3396	0.0060
Waist-hip ratio												
Normal (ref)	—	—	—	—	—	—	—	—	—	—	—	—
Abnormal	−4.0745	−4.6854	−3.4637	<0.0001	−4.8594	−5.9444	−3.7744	<0.0001	−3.1639	−7.4264	1.0985	0.1449
Hypertension												
No (ref)	—	—	—	—	—	—	—	—	—	—	—	—
Yes	−2.4920	−3.4235	−1.5604	<0.0001	−1.7471	−3.3424	−0.1518	0.0319	−4.6206	−11.1030	1.8619	0.1615
LDL-C	0.0076	−0.0014	0.0166	0.0990	−0.0093	−0.0254	0.0069	0.2623	−0.0429	−0.1022	0.0163	0.1548

*β*, regression coefficient; BMI, body mass index; CI, confidence interval; HDL-C, high-density lipoprotein cholesterol; LDL-C, low-density lipoprotein cholesterol; ref, reference group.

Table 5 shows the association between vegetarian diet, rs3764261 genotype, and HDL-C levels, categorized by the joint genotype–diet groups. The reference group consisted of non-vegetarians with the CC genotype. Among non-vegetarians, individuals with the CA (*β* = 3.5413, *p* < 0.0001) and AA (*β* = 8.9435, *p* < 0.0001) genotypes had significantly higher HDL-C levels than those with the CC genotype. Among vegetarians, the CC genotype was associated with significantly lower HDL-C levels (*β* = −2.7082, *p* < 0.0001), while no significant associations were found for the CA (*β* = −1.2350, *p* = 0.0988) or AA (*β* = 1.0128, *p* = 0.6943) genotypes.

**Table 5:** Association of vegetarian diet × rs3764261 joint groups with HDL-C levels.

Variables	*β*	95% CI lower	95% CI upper	*p*-value
Vegetarian diet × rs3764261				
Non-vegetarian, CC (ref)	—	—	—	—
Non-vegetarian, CA	3.5413	2.9709	4.1117	<0.0001
Non-vegetarian, AA	8.9435	7.3270	10.5600	<0.0001
Vegetarian, CC	−2.7082	−3.6651	−1.7514	<0.0001
Vegetarian, CA	−1.2350	−2.7015	0.2314	0.0988
Vegetarian, AA	1.0128	−4.0392	6.0649	0.6943
Gender				
Women (ref)	—	—	—	—
Men	−8.5961	−9.2613	−7.9309	<0.0001
Age, years	0.1093	0.0824	0.1362	<0.0001
Cigarette smoking				
No (ref)	—	—	—	—
Yes	−1.8480	−2.6549	−1.0412	<0.0001
Alcohol consumption				
No (ref)	—	—	—	—
Yes	2.8054	1.7340	3.8768	<0.0001
Exercise habit				
No (ref)	—	—	—	—
Yes	1.2568	0.7399	1.7736	<0.0001
BMI categories				
Normal weight (ref)	—	—	—	—
Underweight	7.0629	5.6713	8.4545	<0.0001
Overweight	−4.8745	−5.4685	−4.2804	<0.0001
Obesity	−7.1065	−7.8223	−6.3907	<0.0001
Waist-hip ratio				
Normal (ref)	—	—	—	—
Abnormal	−4.2921	−4.8232	−3.7611	<0.0001
Hypertension				
No (ref)	—	—	—	—
Yes	−2.2955	−3.0961	−1.4948	<0.0001
LDL-C	0.0019	−0.0059	0.0098	0.6340

*β*, regression coefficient; BMI, body mass index; CI, confidence interval; HDL-C, high-density lipoprotein cholesterol; LDL-C, low-density lipoprotein cholesterol; ref, reference group.

## 4 Discussion

In this large population-based study of 9,263 Taiwanese adults, we identified three principal findings. First, the *CETP* rs3764261 A allele was associated with higher HDL-C levels in an apparent allele-dose manner, with adjusted *β* coefficients of 3.3318 mg/dL for CA and 8.4651 mg/dL for AA carriers compared with the CC genotype (both *p* < 0.0001). Second, a vegetarian diet was independently associated with lower HDL-C concentrations (*β* = −3.4040 mg/dL, *p* < 0.0001). Third, and most importantly, a statistically significant gene–diet interaction was observed between rs3764261 and vegetarian status (*p* = 0.0045). Stratified analyses showed that the positive association between the A allele and HDL-C was confined to non-vegetarians, while the inverse association between a vegetarian diet and HDL-C became progressively stronger across the CC, CA, and AA genotypes (*β* = −2.6779, −4.8479, and −8.1884 mg/dL, respectively). To our knowledge, this is the first study to characterize this specific gene–diet interaction in an East Asian population.

The HDL-C-raising association of the rs3764261 A allele in our non-vegetarian participants is consistent with prior genome-wide association studies and Taiwanese population data linking this variant to higher circulating HDL-C (4, 5, 7, 20). Functional evidence also supports CETP as a biologically plausible determinant of HDL-C, because CETP mediates cholesteryl ester transfer from HDL to apolipoprotein B-containing lipoproteins, and altered CETP protein secretion or activity can affect HDL-C levels. The magnitude of the per-allele effect we observed (approximately 3–4 mg/dL) is broadly comparable to estimates reported in previous Asian and European populations (7, 20), supporting the generalizability of this association. However, the attenuation of this genetic effect among vegetarians has not been previously reported and suggests that the phenotypic expression of rs3764261 is modifiable by dietary context.

The lower HDL-C concentrations observed among vegetarians, despite their more favorable total cholesterol and LDL-C profiles, are consistent with previous observational studies and meta-analyses (9–13). Although vegetarian and vegan diets reliably reduce atherogenic lipoproteins, their net effect on HDL-C has remained inconsistent, with several studies reporting modest decreases (11–13). Proposed mechanisms include lower intake of dietary cholesterol and saturated fat, altered fatty acid composition with reduced long-chain n-3 fatty acids, and shifts in lipid metabolism pathways and the gut microbiome (10, 14). Our findings extend this literature by showing that the magnitude of the vegetarian-associated HDL-C reduction depends on *CETP* genotype: A-allele carriers, who would otherwise have the highest HDL-C levels, exhibited the largest absolute decrement when following a vegetarian diet. A similar pattern of gene–diet modification in HDL-C metabolism has been described in the Taiwan Biobank for the *MTHFR* rs1801133 variant in the context of combined exercise and a vegetarian diet (18), reinforcing the value of nutrigenetic analyses in this cohort.

Several biologically plausible mechanisms may explain the observed interaction. Because CETP participates in HDL remodeling through cholesteryl ester transfer and CETP protein availability (6, 8), the phenotypic effect of rs3764261 may depend on the availability of substrate apolipoprotein B-containing lipoproteins and on the supply of dietary lipids that fuel HDL maturation. Vegetarian diets, which are typically lower in saturated fat and dietary cholesterol and higher in fiber and plant sterols (9, 10, 14), may reduce lipid flux through CETP-dependent pathways and thereby narrow the phenotypic gap between A-allele carriers and CC homozygotes. From a precision-nutrition standpoint, these findings indicate that the cardiometabolic response to a plant-based diet is not uniform across genotypes (15–17). While vegetarian diets remain associated with overall favorable lipid profiles, individuals carrying the *CETP* rs3764261 A allele may experience a disproportionate reduction in HDL-C, a consideration that could inform individualized dietary counseling and risk stratification, particularly in populations with relatively common vegetarian dietary practices, such as Taiwan.

The strengths of this study include its large population-based sample, the use of standardized biochemical measurements and genotyping from the Taiwan Biobank, and adjustment for a comprehensive set of lifestyle and metabolic covariates. Several limitations should also be acknowledged. First, the cross-sectional design precludes causal inference; longitudinal studies are needed to confirm that the observed differences in HDL-C reflect a true effect of a vegetarian diet rather than residual confounding by long-standing lifestyle differences. Second, vegetarian status was self-reported through a questionnaire and did not distinguish among lacto-, ovo-, or strict vegan subtypes, which may have heterogeneous effects on lipid metabolism. Third, although we adjusted for major lipid-related covariates, unmeasured factors such as dietary fatty acid composition, physical activity intensity, and gut microbiome composition were not available and may partially mediate the observed associations. Fourth, only a single *CETP* variant was examined; future studies incorporating multiple *CETP* variants, genetic risk scores, and functional lipoprotein subfractions would provide a more complete picture of the gene–diet interplay. Finally, the relatively small number of AA homozygotes among vegetarians (*n* = 21) limited statistical power for some stratified estimates and warrants replication in independent cohorts.

Future research should build on these findings in several directions. Prospective and interventional studies that use validated dietary assessment and distinguish vegetarian subtypes (lacto-, ovo-, and strict vegan) are needed to establish temporality and to quantify the genotype-specific HDL-C response to plant-based diets. Incorporating multiple CETP variants, polygenic risk scores, and direct measurements of CETP activity and lipoprotein subfractions would help clarify the mechanistic basis of the observed interaction. Replication in independent East Asian and multi-ethnic cohorts—ideally with larger numbers of AA homozygous vegetarians—would improve the precision of the stratified estimates. Finally, linking these gene–diet interactions to hard cardiovascular endpoints will be essential before CETP genotype can be integrated into precision-nutrition recommendations.

## 5 Conclusion

In conclusion, this study provides evidence of a significant interaction between *CETP* rs3764261 and a vegetarian diet on HDL-C levels in a Taiwanese population. The A allele was associated with higher HDL-C primarily among non-vegetarians, and a vegetarian diet was associated with progressively lower HDL-C across the CC, CA, and AA genotypes. These findings support the integration of genetic information into nutritional guidance and underscore the relevance of *CETP* genotype in evaluating the lipid response to plant-based dietary patterns.

## Data Availability

The data that support the findings of this study are available in the Taiwan Biobank. Data access requests should be sent to the corresponding author.
